# Supplementary material for: Periprotein lipidomes of Saccharomyces cerevisiae provide a flexible environment for conformational changes of membrane proteins
Source: eLife. 2020 Apr 17;9:e57003. doi: 10.7554/eLife.57003 (PMC7182430; doi:10.7554/eLife.57003)
Supplement: Supplementary file 1. [file elife-57003-supp1.docx]

**Supplementary tables**

Table S1: All strains and plasmids used in this study

| **Strain/Name** | **Genotype** | **Ref / Origin** |
| --- | --- | --- |
| Y8000 | *ura3-Δ1* | ((15)) *23344C* |
| Y8001/Lyp1 MCP | *ura3-Δ1*, pJK2001 | ((15)) *23344C* |
| Y8002/Can1 MCP | *ura3-Δ1*, pJK2002 | ((15)) *23344C* |
| Y8003/Pma1 MCP | *ura3-Δ1*, pJK2003 | ((15)) *23344C* |
| Y10001/Lyp1 MCC | Sur7p-GBP, Pil1-mCherry, *can1-Δ1, gap1-Δ1, ura3-Δ1*, pJK2001 | ((15)) *SG067* |
| Y10002/Can1 MCC | Sur7p-GBP, Pil1-mCherry, *can1-Δ1, gap1-Δ1, ura3-Δ1*, pJK2002 | ((15)) *SG067* |
| Y5000/Sur7 | *ura3-Δ1*, Sur7p-Ypet | This Study |
| **Plasmid** | **Description** | **origin** |
| pJK2001 | CEN-ARS, pGal_Lyp1p-Ypet_RGShis10, *ura3* | This study |
| pJK2002 | CEN-ARS, pGal_Can1p-Ypet_RGShis10, *ura3* | This study |
| pJK2003 | CEN-ARS, pGal_Pma1p-Ypet_RGShis10, *ura3* | This study |
