# Supplementary material for: Periprotein lipidomes of Saccharomyces cerevisiae provide a flexible environment for conformational changes of membrane proteins
Source: eLife. 2020 Apr 17;9:e57003. doi: 10.7554/eLife.57003 (PMC7182430; doi:10.7554/eLife.57003)
Supplement: Supplementary file 2. — Based on the ions identified from both SMALP-Pma1 and SMALP-Sur7. Other SMALPs: Lyp1-MCP, Lyp1-MCC, Can1-MCP, and Can1-MCC. Values are expressed as pmol lipid per 10 pmol protein. Chromatography peak areas used in our calculations are given for ergosterol and the sphingolipids IPC and MIPC. Phospho- (PC, PI, PE, PS, PG, PA, CL) and sphingolipids (IPC and MIPC) are reported as negative ions, which were shown in Figure 2D. Ergosterol is reported as a positive ion (protonated). [file elife-57003-supp2.docx]

| pmol lipid / 10 pmol protein | | | | | | |
| --- | --- | --- | --- | --- | --- | --- |
| **m/z/lipid chain** | **Pma1 MCP** | **Sur7 MCC** | **Lyp1 MCP** | **Lyp1 MCC** | **Can1 MCP** | **Can1 MCC** |
| **PC** |  |  |  |  |  |  |
| 692.4508/C26:1 | 3.03 ± 0.13 | 0 ± 0 | 2,56 ± 0,49 | 2,45 ± 0,34 | 2.33 ± 0.37 | 1.42 ± 0.32 |
| 722.4977/C28:0 | 1.11 ± 0.13 | 1.06 ± 0.24 | 1,01 ± 0,23 | 0,98 ± 0,2 | 0.98 ± 0.17 | 0.7 ± 0.09 |
| 720.4821/C28:1 | 9.93 ± 0.8 | 2.23 ± 0.47 | 10,52 ± 2,61 | 8,57 ± 2,27 | 8.13 ± 1.47 | 6 ± 0.81 |
| 748.5134/C30:1 | 11.75 ± 1.13 | 2.42 ± 0.5 | 9,19 ± 2,6 | 9,74 ± 2,43 | 10.48 ± 1.82 | 7.41 ± 1.11 |
| 746.4972/C30:2 | 13.38 ± 1.14 | 1.71 ± 0.57 | 10,18 ± 2,08 | 11,01 ± 2,42 | 10.79 ± 1.71 | 8.07 ± 1.11 |
| 776.5447/C32:1 | 18.11 ± 0.69 | 37.82 ± 10.28 | 24,89 ± 9,15 | 16,54 ± 3,04 | 18.09 ± 2.32 | 13.81 ± 1.41 |
| 774.529/C32:2 | 177.7 ± 17.5 | 76.5 ± 19.7 | 160,6 ± 455 | 134,0 ± 32,6 | 159.9 ± 38.9 | 112.5 ± 26.4 |
| 804.576/C34:1 | 15.21 ± 1.45 | 31.44 ± 7.41 | 13,52 ± 4,48 | 13,79 ± 4,38 | 12.76 ± 2.21 | 9.22 ± 2 |
| 802.5603/C34:2 | 101.06 ± 3.49 | 70.8 ± 17.72 | 99,03 ± 28,42 | 76,83 ± 10,1 | 92.97 ± 14.04 | 66.37 ± 14.55 |
| 832.6073/C36:1 | 1.98 ± 0.18 | 5.68 ± 2 | 3 ± 1,17 | 1,63 ± 0,19 | 2.22 ± 0.79 | 1.35 ± 0.46 |
| 830.5916/C36:2 | 6.62 ± 1.27 | 7.01 ± 2.29 | 11,88 ± 6,78 | 4,96 ± 1,68 | 6.15 ± 1.99 | 5.28 ± 2.26 |
| **PI** |  |  |  |  |  |  |
| 725.4246/C26:0 | 1.98 ± 0.28 | 0.44 ± 0.14 | 1,65 ± 0,46 | 1,81 ± 0,46 | 1.97 ± 0.56 | 1.12 ± 0.28 |
| 753.4559/C28:0 | 5.92 ± 0.71 | 1.47 ± 0.38 | 4,58 ± 1,13 | 4,57 ± 1,24 | 5.25 ± 1.32 | 3.31 ± 0.69 |
| 751.4403/C28:1 | 1.4 ± 0.15 | 0 ± 0 | 0,8 ± 0,2 | 1,01 ± 0,24 | 0.97 ± 0.24 | 0.61 ± 0.16 |
| 779.4716/C30:1 | 7.48 ± 1.04 | 0 ± 0 | 4,49 ± 1,02 | 5,75 ± 1,73 | 5.09 ± 1.66 | 4.03 ± 1 |
| 807.5029/C32:1 | 37.64 ± 4.87 | 18.31 ± 4.05 | 34,09 ± 9,66 | 28,14 ± 8,04 | 34.62 ± 7.99 | 23.3 ± 5.7 |
| 805.4872/C32:2 | 13.04 ± 1.24 | 5.23 ± 1.23 | 10,31 ± 2,33 | 9,22 ± 2,01 | 10.09 ± 1.75 | 7.35 ± 1.64 |
| 835.5342/C34:1 | 58.15 ± 7.45 | 40.37 ± 11.86 | 55,76 ± 18,5 | 41,09 ± 10,71 | 55.14 ± 13.58 | 38.64 ± 9.53 |
| 833.5185/C34:2 | 14.73 ± 1.53 | 7.9 ± 2.61 | 13,1 ± 4,06 | 10,33 ± 2,51 | 13.11 ± 3.06 | 9.28 ± 2.19 |
| 863.5655/C36:1 | 15.74 ± 1.54 | 10.89 ± 3.98 | 15,29 ± 5,58 | 10,96 ± 2,52 | 14.44 ± 3.27 | 10.8 ± 2.35 |
| 861.5498/C36:2 | 1.37 ± 0.06 | 0.99 ± 0.31 | 1,26 ± 0,45 | 0,9 ± 0,2 | 1.18 ± 0.24 | 0.92 ± 0.19 |
| **PE** |  |  |  |  |  |  |
| 606.414/C26:0 | 0.65 ± 0.01 | 0 ± 0 | 0 ± 0 | 0 ± 0 | 0 ± 0 | 0 ± 0 |
| 634.4453/C28:0 | 0.73 ± 0.02 | 0 ± 0 | 0,56 ± 0,13 | 0,49 ± 0,12 | 0.54 ± 0.06 | 0.47 ± 0.07 |
| 632.4296/C28:1 | 3.57 ± 0.4 | 0 ± 0 | 2,6 ± 0,61 | 2,77 ± 0,89 | 3.11 ± 0.42 | 2.23 ± 0.49 |
| 660.4609/C30:1 | 4.9 ± 0.47 | 0 ± 0 | 3,06 ± 0,69 | 3,62 ± 1,06 | 4 ± 0.43 | 3.03 ± 0.55 |
| 658.4453/C30:2 | 2.2 ± 0.4 | 0 ± 0 | 1,17 ± 0,28 | 1,61 ± 0,57 | 1.9 ± 0.26 | 1.35 ± 0.34 |
| 688.4922/C32:1 | 19.14 ± 1.23 | 5.41 ± 1.61 | 16,34 ± 3,8 | 13,46 ± 3,04 | 14.88 ± 1.84 | 12.48 ± 2.44 |
| 686.4766/C32:2 | 37.88 ± 6.66 | 17.38 ± 3.42 | 29,59 ± 6,36 | 29,36 ± 8,28 | 35.51 ± 4.26 | 26.77 ± 6.61 |
| 716.5235/C34:1 | 16.34 ± 1.65 | 5.97 ± 1.94 | 13 ± 4,72 | 10,18 ± 2,83 | 10.59 ± 1.5 | 10.15 ± 1.23 |
| 714.5079/C34:2 | 58.31 ± 7.03 | 23.42 ± 5.13 | 50,17 ± 12,15 | 43,34 ± 9,29 | 57.73 ± 8.83 | 43.95 ± 10.36 |
| 742.5392/C36:2 | 13.33 ± 4.01 | 4.74 ± 2.92 | 35 ± 27,9 | 10,07 ± 5,34 | 12.35 ± 5.41 | 11.95 ± 7.86 |
| **PS** |  |  |  |  |  |  |
| 732.4821/C32:1 | 24.41 ± 2.05 | 9.66 ± 2.03 | 15,08 ± 4,08 | 16,61 ± 4,51 | 18.06 ± 2.52 | 12.68 ± 3 |
| 730.4664/C32:2 | 21.59 ± 2.16 | 12.63 ± 2.03 | 16,25 ± 4,36 | 18,05 ± 5,27 | 21.47 ± 4.31 | 13.24 ± 4.21 |
| 760.5134/C34:1 | 29.86 ± 1.69 | 21.4 ± 7.29 | 19,22 ± 6,22 | 19,11 ± 4,33 | 21.8 ± 2.91 | 15.24 ± 3.5 |
| 758.4977/C34:2 | 68.04 ± 6.34 | 63.41 ± 16.1 | 52,99 ± 15,47 | 51,66 ± 13,49 | 67.27 ± 12.28 | 43.25 ± 12.91 |
| 788.5447/C36:1 | 1.9 ± 0.08 | 2.69 ± 0.43 | 1,9 ± 0,6 | 1,24 ± 0,12 | 0 ± 0 | 0 ± 0 |
| **PG** |  |  |  |  |  |  |
| 719.4868/C32:1 | 0.84 ± 0.33 | 0 ± 0 | 1.73 ± 0.79 | 0.71 ± 0.5 | 0.72 ± 0.42 | 0.18 ± 0.06 |
| 747.5181/C34:1 | 0.95 ± 0.31 | 0 ± 0 | 1.73 ± 0.77 | 0.75 ± 0.47 | 0.82 ± 0.45 | 0.3 ± 0.12 |
| **PA** |  |  |  |  |  |  |
| 643.4344/C32:2 | 3.24 ± 0.45 | 0 ± 0 | 2.04 ± 0.87 | 1.96 ± 0.79 | 2.72 ± 0.91 | 1.67 ± 0.7 |
| 671.4657/C34:2 | 3.2 ± 0.28 | 0 ± 0 | 3.09 ± 1.37 | 1.9 ± 0.63 | 2.83 ± 0.83 | 2 ± 0.7 |
| **CL** |  |  |  |  |  |  |
| 1261.8241/C58:3 | 0.62 ± 0.05 | 0 ± 0 | 0.5 ± 0.18 | 0.42 ± 0.12 | 0.5 ± 0.09 | 0.33 ± 0.05 |
| 1263.8397/C58:2 | 0.54 ± 0.03 | 0 ± 0 | 0 ± 0 | 0 ± 0 | 0.31 ± 0.02 | 0.26 ± 0.06 |
| 1289.8554/C60:3 | 1.35 ± 0.12 | 0 ± 0 | 1.24 ± 0.45 | 0.89 ± 0.3 | 1.07 ± 0.27 | 0.76 ± 0.17 |
| 1291.871/C60:2 | 0.84 ± 0.16 | 0 ± 0 | 1.08 ± 0.38 | 0.5 ± 0.04 | 0.85 ± 0.18 | 0.44 ± 0.1 |
| 1317.8867/C62:3 | 1.22 ± 0.12 | 0 ± 0 | 1.08 ± 0.4 | 0.78 ± 0.26 | 0.94 ± 0.26 | 0.65 ± 0.16 |
| 1343.9023/C64:4 | 1.39 ± 0.14 | 0 ± 0 | 1.3 ± 0.48 | 0.89 ± 0.32 | 1.1 ± 0.28 | 0.72 ± 0.17 |
| 1371.9336/C66:4 | 2.56 ± 0.27 | 0 ± 0 | 2.7 ± 1 | 1.73 ± 0.58 | 2.12 ± 0.57 | 1.46 ± 0.37 |
| 1399.9649/C68:4 | 2.33 ± 0.23 | 0.5 ± 0.21 | 2.39 ± 0.92 | 1.56 ± 0.48 | 1.92 ± 0.58 | 1.33 ± 0.33 |
| 1427.9962/C70:4 | 0.88 ± 0.1 | 0 ± 0 | 0.89 ± 0.32 | 0.59 ± 0.15 | 0.72 ± 0.23 | 0.52 ± 0.13 |
| **ergosterol** |  |  |  |  |  |  |
| 397.3459 | 28.17 ± 5.61 | 24.45 ± 4.61 | 11.95 ± 6.15 | 20 ± 12.93 | 22.54 ± 5.98 | 27.65 ± 6.06 |
| **Chromatography peak areas** | | | | | | |
| **ergosterol** |  |  |  |  |  |  |
| 397.3459 | 44390 ± 7358 | 39465 ± 6204 | 31142 ± 18607 | 21443 ± 9362 | 36755 ± 8084 | 43658 ± 8191 |
| **IPC** |  |  |  |  |  |  |
| 952.6846/C42:0 | 23809 ± 3506 | 6679 ± 2040 | 21481 ± 7033 | 12324 ± 5434 | 9134 ± 2505 | 10023 ± 2452 |
| **MIPC** |  |  |  |  |  |  |
| 1114.735/C42:0 | 14005 ± 3680 | 2797 ± 697 | 16561 ± 6280 | 6231 ± 2545 | 5020 ± 2244 | 4784 ± 944 |
